# The Role of FAT10 in Alcoholic Hepatitis Pathogenesis

**DOI:** 10.3390/biomedicines8070189

**Published:** 2020-07-01

**Authors:** Yue Jia, Ping Ji, Samuel W. French

**Affiliations:** Harbor-UCLA Medical Center, Torrance, CA 90509, USA; yjia@dhs.lacounty.gov (Y.J.); sfrench@dhs.lacounty.gov (S.W.F.)

**Keywords:** FAT10, alcoholic hepatitis, pathogenesis, alcohol toxicity, molecular mechanisms, low-dose effects

## Abstract

FAT10 expression is highly up-regulated by pro-inflammatory cytokines IFNγ and TNFα in all cell types and tissues. Increased FAT10 expression may induce increasing mitotic non-disjunction and chromosome instability, leading to tumorigenesis. In this review, we summarized others’ and our work on FAT10 expression in liver biopsy samples from patients with alcoholic hepatitis (AH). FAT10 is essential to maintain the function of liver cell protein quality control and Mallory–Denk body (MDB) formation. FAT10 overexpression in AH leads to balloon degeneration and MDB aggregation formation, all of which is prevented in fat10-/- mice. FAT10 causes the proteins’ accumulation, overexpression, and forming MDBs through modulating 26s proteasome’s proteases. The pathway that increases FAT10 expression includes TNFα/IFNγ and the interferon sequence response element (ISRE), followed by NFκB and STAT3, which were all up-regulated in AH. FAT10 was only reported in human and mouse specimens but plays critical role for the development of alcoholic hepatitis. Flavanone derivatives of milk thistle inhibit TNFα/IFNγ, NFκB, and STAT3, then inhibit the expression of FAT10. NFκB is the key nodal hub of the IFNα/TNFα-response genes. Studies on Silibinin and other milk thistle derivatives to treat AH confirms that overexpressed FAT10 is the major key molecule in these networks.

## 1. Introduction

In developed countries, it’s reported that the toxicity of alcohol is dose-related at an individual level and at a population level, as reported in different European countries over the last few decades. Overall liver mortality is largely determined by population alcohol consumption [1]. Chronic alcohol use leads to alcoholic liver disease (ALD), including three major disease conditions: alcoholic fatty liver, alcoholic hepatitis, and cirrhosis. Alcohol content varies between different types of beverages; a standard drink is defined as 14 g, or 18 mL, or 0.6 fl oz of pure alcohol in the USA. About three decades ago, Sorensen summarized that alcohol abuse has a permissive—rather than a dose-dependent—role in the development of alcoholic liver injury [2]. A prospective monitoring of alcohol consumption, studying the rate of development of cirrhosis per unit of time, could be the ideal study design, but is not feasible. It is not well established what is the low-dose effect of alcohol in liver clinically, morphologically, or molecularly. We studied the specimens from diagnosed alcohol hepatitis patients and control groups to explore the possible molecular target(s) for the alcohol hepatitis pathogenesis. We summarized our and other researchers’ work to present the human leukocyte antigen (HLA)-F-adjacent transcript 10 (FAT10, also known as ubiquitin D (UBD)) and explain that FAT10 may be useful to study the spectrum of alcohol-related liver diseases, including finding out what is the low-dose effect of alcohol in liver.

FAT10 is a ubiquitin-like modifier that directly targets proteins for proteasomal degradation. The post-translational modification by ubiquitin (Ub) or Ub-like (UBL) modifiers is related with almost all eukaryotic cell functions. Human leukocyte antigen-F adjacent transcript 10 (FAT10) is a member of UBL modifiers only found in mammals [3]. FAT is expressed in the immune tissues [4,5,6], or other cell types, upon inflammation [7,8]. Importantly, FAT10 is commonly overexpressed in numerous types of cancer [9,10].

In previous studies, FAT10, when expressed in high levels, has been shown to result in increased mitotic non-disjunction and chromosome instability, leading to tumorigenesis [11]. FAT10 expression is highly up-regulated by pro-inflammatory cytokines IFNγ and TNFα in all cell types and tissues [10]. It interacts with and influences downstream targets, such as MAD2, p53, or β-catenin, leading to enhanced survival, proliferation, invasion, and metastasis of cancer, as well as non-malignant cells [10]. FAT10 is an oncogene that is associated with cellular malignancy, probably through its interaction with mitotic arrest-deficient 2 (MAD2) [12]. However, other FAT10 pathways also lead to tumorigenesis, such as NFκB, Wnt, and SMAD2 signaling [10]. FAT10 up-regulation is critical for GRP78-mediated hepatocellular carcinoma (HCC) proliferation. GRP78 modulates FAT10 expression via the NFκB signal pathway [13]. The FAT10 gene expression is up-regulated in 90% of HCCs [5].

## 2. Role of FAT10 in Alcoholic Liver

**Figure 1:**
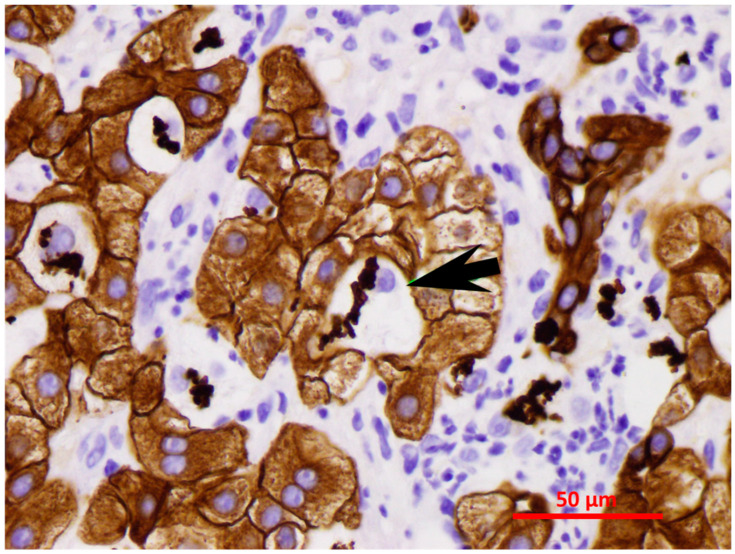
MDBs in balloon cells (CAM5.2 IHC staining, ×640). The black arrow points to the balloon cells with Mallory–Denk bodies (MDBs). MDBs and bile duct metaplasia are brown in color, with CAM5.2 IHC staining. The lymphocytes (CD4+) are nibbling the hepatocytes at the MHC antigen binding sites (immunologic synapsis) to gradually remove the hepatocytes [14].

FAT10 expression was measured in clinical trial-derived liver biopsies from patients with clinical evidence of alcoholic hepatitis (Table 1, unpublished primary data). Due to lack of a generally accepted histopathology score system for alcoholic hepatitis, we established our own score system for human alcoholic liver disease specimens. In brief, fat macrovesicular or microvesicular deposition and Mallory–Denk bodies (MDBs) are scored from 0 to 4 (0: no fat deposition or MDB formation; 1: ≤25%; 2: ≤50%; 3: ≤75%; 4: >75%), fibrosis stage from 0 to 4 (0: none; 1: perisinusoidal; 2: periportal ± perisinusoidal; 3: bridging fibrosis; 4: cirrhosis), while polymorphonuclear leukocyte (PMN) and lymphocytes (lymp) are scored from 0 to 4 (0: none; 1: focal lobular; 2: focal portal; 3: lobular and portal; 4: all three zones). Morphologic features of alcoholic hepatitis (AH) balloon cells forming MDBs and bile duct metaplasia stained with CAM5.2 IHC staining are shown in Figure 1 [14]. The CAM5.2 predilute antibody was purchased from a commercial organization (Cat# 790-4555, Ventana Medical system, Roche, Pleasanton, CA) and the working concentration was 11 microgram/mL. The ultraView Universal DAB Detection Kit was used for detecting mouse CAM5.2 primary antibody (Cat# 760-500, Ventana Medical system, Roche, Pleasanton, CA). The kit is intended to identify targets by immunohistochemistry (IHC) in sections of formalin-fixed paraffin-embedded tissue. The specimen was formalin-fixed paraffin-embedded and cut at 5-micrometer thickness. These two morphologic features are consistently found in AH. In alcoholic liver, FAT10 is essential to maintain the functional quality control of liver cell proteins and to modulate MDB formation. This is supported by experiments with FAT10 knockout (KO) mice (*fat10-/-*), which fail to form MDBs [14].

### 2.1. Role of MDB and Its Modulation in Animal Studies

In general, Mallory–Denk body formation presents in liver diseases including alcoholic liver disease (ASH), fatty liver in obesity (NASH), HCV, hepatocellular carcinoma, and others [15,16]. Ubiquitin, intermediate filament proteins keratins 8 and 18 (K8/18), and P62/sequestosome-1 are the main elements presented in a Mallory body [17]

DDC-feeding causes the induction of the catalytic subunits of the immunoproteasome in mice. At the same time, DDC-feeding decreases the expression of the catalytic subunits of the 26S proteasome. In previous work, mice fed DDC and SAMe showed markedly reduced MDB formation in vivo [18,19]. The molecular mechanism of the inhibition of MDB formation caused by SAMe was achieved via preventing the DDC-feeding-induced inhibition of the 26S proteasome activity. Other researcher’s work proved that the inhibition of MDB formation caused by SAMe was achieved through blocking the 26S proteasome switching to the immunoproteasome [18]. DDC-feeding also regulates FAT10, TNFα, and IFNγ receptors. The interferon sequence response element (ISRE) is located on the FAT10 promoter. The ISRE responds to IFNγ to induce the immunoproteasome catalytic subunits LMP2 and LMP7 [9]. SAMe feeding reversed these changes and restored the proteasome to normal, despite DDC-feeding [18].

Betaine feeding also inhibited MDB formation by increasing MATIα expression [20]. It has been reported [21] that betaine, a promethylating agent and methyl donor with strong anti-oxidant properties, can prevent MDB formation via restoring the down-regulation of the Ufm1 conjugation pathway in the livers of DDC re-fed mice. This suggested that MDB formation can be modulated epigenetically. It was reported that promoter methylation is well-correlated with transcriptional silencing of Ufmylation in multiple AH and NASH biopsy samples with MDBs formation. There is clinical evidence of direct involvement of the Ufm1 conjugation system with promoter CpG methylation in liver MDB formation.

Lastly, our previous studies showed that FAT10 KO mice fed DDC failed to develop MDBs. This phenomenon may be due to the 26s proteasomes retaining the normal proteasomal chymotrypsin proteolytic activity without the FAT10 promoter present, even when DDC was fed. MDBs form when the 26s proteasome is suppressed in these FAT10 KO mice model. In contrast, MDBs cannot form if the 26s proteasome is not suppressed [19,22].

The ubiquitin-fold modifier 1 (Ufm1) is a key post-translational modifier which is a member of the ubiquitin-like protein (UBL) family. The Ufmylation plays important roles in endoplasmic reticulum (ER) homeostasis [23,24] and cell cycle control [25,26]. The Ufmylation is also involved in various diseases, including AH, NASH, and liver cancer [23,25,27]. MDB formation may down-regulate the Ufm1 conjugation via hypermethylation of the promoter CpG in AH and NASH patients. The maintenance of Ufmylation methylation may be mediated by DNMT1 and DNMT3B together, which would explain the down-regulation of Ufmylation observed in ASH and NASH liver biopsies [21].

### 2.2. Role of FAT10 and MDB in Human Studies

FAT10 promotes carcinogenesis by directly increasing the survival, proliferation, invasion, and metastasis formation of tumor cells. Liver cancer is the second leading cause of cancer related deaths worldwide [28]. The major risk factors include alcoholism, viral hepatitis (HBV & HCV), and obesity/metabolic syndrome, which all may progress to hepatocellular carcinoma (HCC). Alcoholic liver disease (ALD) and AH are the major causes of HCC in the US [29,30,31]. AH may progress to fibrosis, cirrhosis, and ultimately hepatocellular carcinoma (HCC); about 10–20% of ASH cases progress to cirrhosis [32,33] and 7–10% to HCC [32], annually. ALD is the most common cause of HCC, which accounts for around one third of all HCC cases in the US and Italy [29,31]. It is believed that alcohol is a factor in the development of HCC through the genotoxic (direct) pathway and cirrhosis (indirect) mechanisms. The molecular mechanisms by which ASH progress to HCC are not well established and there are many theories which have been postulated to explain this, including the loss of tumor suppressor genes, altered DNA methylation, genetic susceptibility, oxidative stress, and decreased liver retinoic acid level [29,34,35]. In our previous studies, we showed that, in addition to the TLR/NFKB/CXCR4/7 and PI3K/AKT/mTORC1 signaling pathways, the Tec kinase signaling pathway connects these two systems together in Mallory–Denk body (MDB) formation in AH. In these studies, we proved that FAT10 [36,37,38], plays an important role in MDB formation and tumorigenesis as a proteasomal degradation signal [9,39,40].

**Figure 2:**
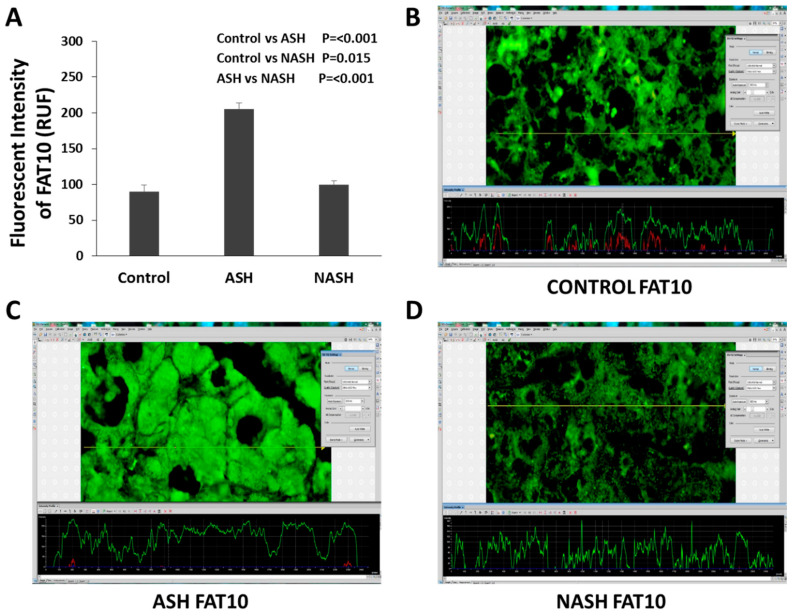
Different changes of FAT10 in ASH and NASH specimens. (**A**) Level of expression of FAT10 protein up-regulated in ASH, compared with NASH or normal controls. Expression is measured as fluorescence intensity and displayed as mean +/− standard deviation. Representative images of fluorescence intensity to measure FAT10 expression in normal control (**B**), ASH (**C**), and NASH (**D**) liver specimens. A line is drawn through the image to yield a fluorescence intensity graph; the intensity of the ten highest peaks are measured. Three areas per slide are measured in this manner including Figure 2 and Figure 3. FAT10 is sequestered within MDBs in AH, suggesting that the excess expression of FAT10 in AH is leading to the stabilization of the protein due to loss of protein quality control. In fluorescent assays, fluorescence intensity is not an absolute measurement and is usually quantified in relative fluorescence units (RFU) [41].

The liver biopsies from patients with alcoholic hepatitis were studied. By measuring the amount of protein with fluorescent intensity staining, the total proteins, including the FAT10 stabilized proteins, were quantitated. Using this technology, we can measure the amount of each protein in the control livers, the AH livers, and the non-alcoholic steatohepatitis (NASH) livers simultaneously, for comparison. Figure 2 shows the immunofluorescence results of the FAT10 protein, where the liver cell cytoplasm and nucleus of the hepatocytes are filled with FAT10 [41]. FAT10 is sequestered within MDBs in alcoholic steatohepatitis (ASH), suggesting that the excess expression of FAT10 in ASH is leading to the stabilization of the protein. Changes of FAT10 in ASH and NASH specimens are different. FAT10 protein expression is up-regulated in ASH but not in NASH or normal controls (Figure 2A) which indicates the specific effect of FAT10 in ASH. These results indicate that FAT10 increased in the ASH, compared with control group, and even compared with the NASH specimens [41].

### 2.3. Interaction of FAT10 and SUMO

Post-translational protein modification by the small ubiquitin-like modifier (SUMO), termed SUMOylation, is an important mechanism in cellular responses to stress and one that appears to be up-regulated in many cancers. Similar to ubiquitin, SUMOylation regulates protein localization and activity as a critical molecular pathway in cells [42]. As a reversible post-translational modulator, SUMOylation plays an important role in immune response regulating, DNA damage repairing, cell cycle progression, apoptotic modification, and carcinogenesis [42]. There are four SUMO isoforms, which have been reported as SUMO1, SUMO2/3, and SUMO4. The works of other researchers confirmed that the SUMO molecular pathway is identified in all eukaryotes and is involved in genomic integrity maintenance and gene expression modulation, as well as intra-cellular signaling [43]. Alcohol-inducible enzyme Cytochrome P450 (CYP2E1) metabolizes alcohol-producing toxic reactive oxygen species (ROS) and is regulated at the post-translational level. Alcohol-mediated up-regulation of CYP2E1 via SUMOylation, enhancing its protein stability and activity, may be associated with the development of alcoholic liver disease (ALD) [44].

In addition to binding to hundreds of substrate proteins covalently and leading to their rapid degradation independently of ubiquitylation, FAT10 may modulate the activation of SUMO1/2/3 in vitro and down-regulates SUMO conjugation and the SUMO-dependent formation of promyelocytic leukemia protein (PML) bodies in cells. It has been showed that FAT10 directly binds to and impedes the activity of the heterodimeric SUMO E1-activating enzyme AOS1/UBA2 by competing with SUMO for activation and thioester formation. Furthermore, activation of FAT10 by AOS1/UBA2 does not lead to covalent conjugation of FAT10 with substrate proteins which rely on its cognate E1 enzyme, UBA6 [45].

### 2.4. Modulation of Proteasome by FAT10

As above-mentioned, FAT10 can target proteins for degradation. FAT10 is synergistically induced by IFNγ and TNFα in liver parenchymal cells. Previously, we investigated whether FAT10 in liver cells is activated by the innate immunity factor, IFNα, and how alcohol-induced oxidative stress affects the level of FAT10 in liver cells. HCV(+) transgenic mice that express structural HCV proteins and their HCV(-) littermates were fed the De Carli diet (control and alcohol). Alcohol intake induced steatosis and oxidative stress but decreased proteasome activity in the livers of these mice, with more robust response to alcohol in HCV(+) mice [46]. The transcriptional activation of FAT10 induced by IFNα in liver cells was dysregulated by alcohol feeding. IFNα-induced expression of FAT10 in hepatocytes was also suppressed by alcohol exposure in both HCV(+) and HCV(-) mice. This suppressive procedure was accompanied with alcohol-induced lipid peroxidation. FAT10 may target oxidation-related proteins for proteasomal degradation, and the reduced FAT10 levels, along with decreased proteasome activity, may contribute to the stabilization of these altered proteins in hepatocytes. Betaine-feeding reverses alcohol with or without HCV-induced dysregulation of protein methylation and oxidative stress, thereby restoring the FAT10 expression on liver cells [46].

The transcript regulation of Ufmylation and FATylation for protein quality control is down-regulated in the livers of DDC re-fed mice forming Mallory–Denk bodies (MDBs). This is also found in the livers of patients who form MDBs [21]. It has been reported in the literature that, in liver biopsy samples from patients with active chronic hepatitis or cirrhosis, the immunoproteasome formation increased and was proportionally associated with the activity of chronic inflammation in both the cytoplasm and nuclei of the hepatocytes. In biopsy samples from patients with steatohepatitis and control groups, FAT10, LMP2/LMP7, and MECL-1 co-localization was found in the mitochondria in normal liver, while only LMP2 and LMP7 expressions were detected in the cell nuclei, in spite of the formation of Mallory–Denk bodies (MDBs). Meanwhile, in the liver biopsy samples from patients suffering from steatohepatitis with MDB formation, the co-localization of FAT10 and ubiquitin with LMP2, LMP7, and MECL-1 was identified within the MDB. These findings suggest that the immunoproteasome is involved in MDB formation, in samples from steatohepatitis patients. The increase in the immunoproteasome subunit proteins was made at the expense of the 26S proteasome. This indicates that the shift from the 26S to the immunoproteasome had occurred in the MDB-positive hepatocytes [47].

Based on the published data, FAT10 was only found in human and mouse liver specimens and plays critical role in the development of alcoholic hepatitis. The interferon sequence response element (ISRE) is located on the FAT10 promoter and responds to IFNγ to induce the immunoproteasome catalytic subunits LMP2 and LMP7, which leads to colocalization of FAT10/ubiquitin with LMP2, LMP7 to modulate the formation of MDB. To study the low-dose alcohol effect on liver, the changes in transcription, epigenetic modulation, translation, and post-translation modulation levels could be the target parameters. FAT10 is involved in multiple systems and different molecular levels, which makes itself a potential target in related research.

## 3. Molecular Pathways of FAT10 in Liver

It has been accepted that constitutive induction of FAT10 has deleterious consequences in cellular malignancy development [48]. Furthermore, up-regulated FAT10 was reported in several tumor types, including tumors of the liver and colon [6,9,49,50]. Many researchers reported that the increased level of FAT10 associated with Akt, NF-κB/STAT3 [48,51], p53 [8], CXCR4/7 [48], β-catenin/TCF4 [10], and MAD2 [52], in addition to its signal for proteasomal degradation [53].

### 3.1. Studies in FAT10 Knockout Animal

**Figure 3:**
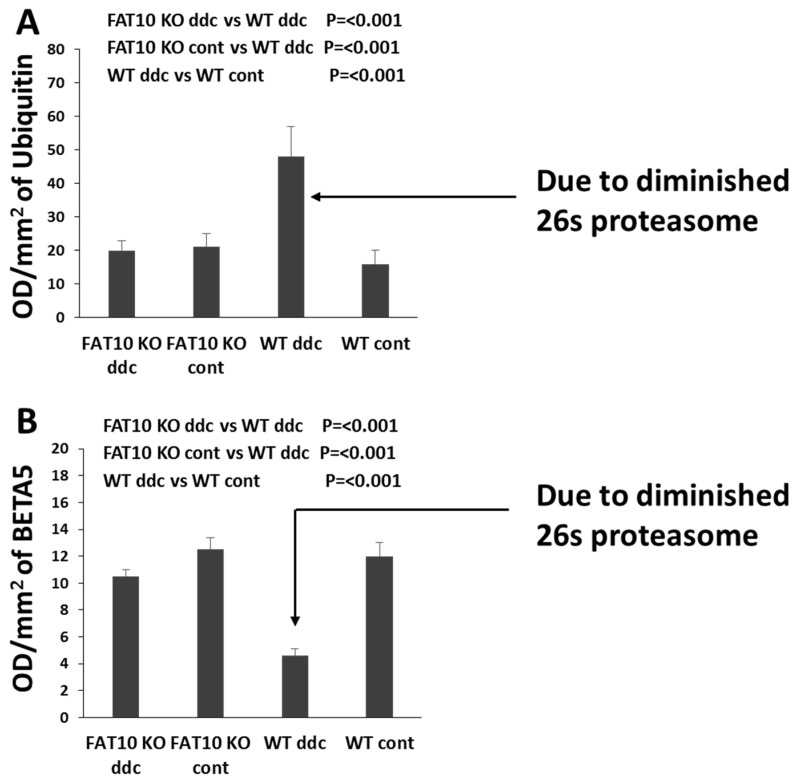
The changes of 26s proteasome in wild type and FAT10 KO mice. (**A**) The FAT10 KO mice fed DDC did not develop the decrease in the 26S proteasome, whereas the wild type DDC fed did. Consequently, digestion of polyubiquinated proteins to form aggresomes (MDBs) did not occur in the FAT10 KO mice. (**B**) Chymotrypsin was diminished in the wild type DDC fed group. In the other groups it was not diminished. This indicates that FAT10 WD mice fed DDC were prevented from forming a decrease in the 26S proteasome catalytic subunit because of the absence of the FAT10-dependent decrease in the expression of BETA5 [14].

**Figure 4:**
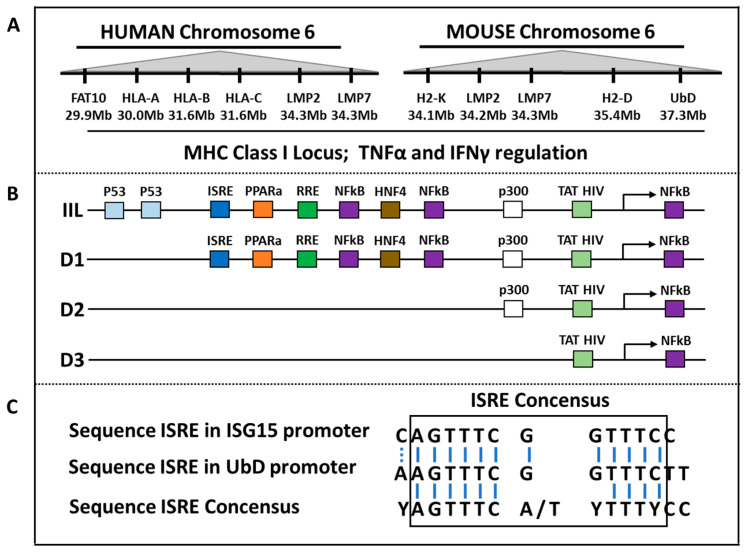
Cloning of FAT10 promoter region. (**A**) Comparison of the histocompatibility MHCI locus between human and mouse. (**B**) Scheme of mouse UbD Promoter. The size of the cloned UbD promoter is 4.2 Kb. p53 down-regulating UbD expression while TAT HIV sequence has been found to be involved in the up-regulation of UbD. One ISRE consensus sequence was found in the promoter at 3.5 Kb upstream from the starting point. (**C**) Comparison of the UbD ISRE, ISG15 ISRE and the consensus ISRE is shown [9].

The mechanism that is responsible for the loss of the ability to form MDBs is found in the FAT10 promoter in humans and mice where the interferon sequence response element (ISRE) is located [8,9] (Figure 4). Several genes are expressed here, including NFκB, in response to the up-regulation by TNFα and IFNγ [10,12]. FAT10 also signals a switch in the 26s proteasome’s three proteases to replace them with the three immunoproteases (MEK-1, LMP2, and LMP7) [9]. This causes a failure to turnover proteins in a timely manner, leading to their accumulation and stabilization, but are ubiquitinated and apparently overexpressed [9,14,41,46] (Figure 3). This leads the stabilized proteins to form aggresomes, in the form of MDBs, in response to the overexpression of IFNγ and TNFα [46]. The increase in ubiquitinated protein is demonstrated by the Western blot (Figure 3A). This reduction of proteolysis by the 26S proteasome in *fat10-/-* mice is demonstrated by a diminished rate of proteolysis in the livers of mice, compared to the control wild type mice 26S proteasome chymotrypsin activity, measured by the Western blot method (Figure 3B). This stabilization of proteins adds to the length of time of turnover and the apparent overexpression of proteins, including cytochrome P450 2E1 (CYP2E1), hydroxynonenal (HNE), and glutathione S-transferase (GST), which leads to oxidative stress when alcohol is consumed.

FAT10 overexpression in AH causes an extensive change in protein content in the liver, leading to balloon degeneration and MDB aggregation formation, all of which is prevented in *fat10-/-* mice [14]. The expression of FAT10 was increased 4.5-fold in the AH livers, but not in the NASH or control livers (Figure 2B–D).

### 3.2. FAT10 and Ubiquitin-Proteasome System (UPS)

Many proteins are degraded by the lysosomes. While outside of the lysosome, most short- or long-lived proteins are degraded through the “ubiquitin-proteasome system (UPS)”, in a living cell. It has been shown that the 26S proteasome and the cylindrical 20S proteasome, which is the key proteolytic site of the UPS, cannot efficiently nor significantly degrade unmodified proteins, so protein substrates of the UPS have to be labeled with a transferring signal peptide in order to be able to bind the proteasome tightly and be transferred for degradation. As a small protein tag, ubiquitin binds the protein candidates via post-translational modification and facilitates the proteasomal degradation of the proteins [53]. Binding with a single ubiquitin is not enough for a modifiable substrate protein to achieve degradation by the 26S proteasome. To perform the signal function, at least four or more ubiquitin molecules must be assembled as a “ubiquitin chain” upon the substrate, or preformed chains must be transferred onto the substrate [54].

FAT10 may function as a second transferable signal (in addition to ubiquitin) for protein candidates to be degraded via the 26S proteasome procedure. It is very interesting that FAT10 is the only ubiquitin-like modifier which may directly transfer the protein substrates to be degraded by the 26S proteasome. In addition, FAT10 directly targets and binds to the 26S proteasome and does not need secondary molecular polymer formation or ubiquitin-chain to play the role of a transfer tag for degradation; this is the reason that FAT10 and 26S proteasome are also known as the FAT10-proteasome system (FPS). FAT10 contains two ubiquitin-like domains, which are sufficient for the transferring and targeting, while four or more molecules of ubiquitin are needed to help protein substrates degradation by the proteasome efficiently [54]. However, Ciechanover and colleagues reported that ubiquitylation of FAT10 enhances the rate of FAT10-mediated degradation [55]. Whether ubiquitylation of FAT10 accelerates FAT10-mediated protein destruction or not remains controversial. Further experiments are needed to settle this remaining issue.

**Figure 5:**
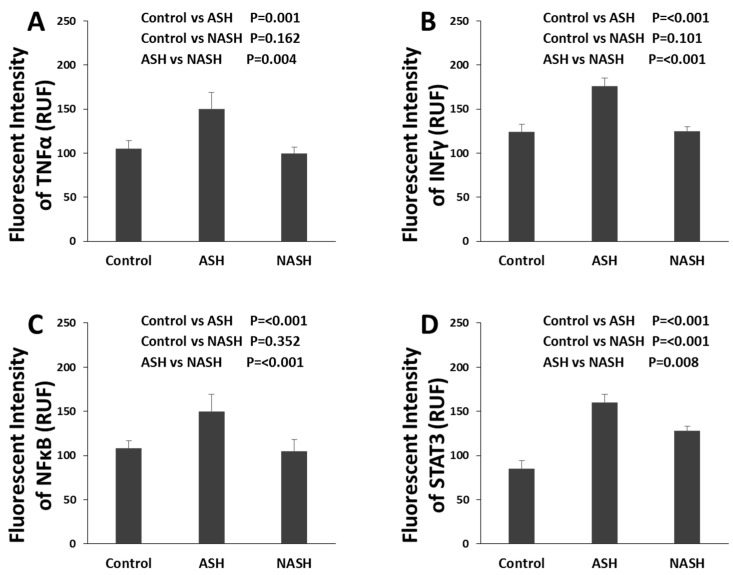
Different changes of molecules in ASH and NASH specimens. (**A**) Level of expression of TNFα protein up-regulated in ASH but not in NASH, compared with normal controls (both *p* < 0.001). Expression is measured as fluorescence intensity and displayed as mean +/− standard deviation in this Figure 5A–D. (**B**) Level of expression of INFγ protein up-regulated in ASH but not in NASH compared with normal controls (both *p* < 0.001). (**C**) Level of expression of NFκB protein up-regulated in ASH but not in NASH compared with normal controls (both *p* < 0.001). (**D**) Level of expression of STAT3 protein up-regulated in ASH and NASH compared with normal controls (both *p* < 0.001), but also significantly higher in ASH compared with NASH (*p* = 0.008) [9,21,23].

FATylation is catalyzed by an activating E1 enzyme, UBA6, and a conjugating E2 enzyme, UBA6-specific enzyme 1 (USE1), both of which carry a conserved catalytic residue to form a thioester intermediate with the FAT10 C-terminus [53]. Whether FAT1ylation requires an E3 enzyme in vivo, as Ub and other UBL modifiers do, remains unknown. The pathway that increases FAT10 expression includes TNFα and IFNγ, followed by NFκB and STAT3, all of which were up-regulated in alcoholic hepatitis (Figure 5A–D) [9,21,23]. Other proteins in the Fatylation pathway (Utcl, Ufml, Uba5, and Uba6) were significantly decreased [21]. There is a di-Gly motif at the C-terminus of FAT10, which is a key structure for the covalent attachment of Ub family modifiers to substrates via a conserved enzyme cascade pathway.

### 3.3. FAT10 and Cytokines

**Figure 6:**
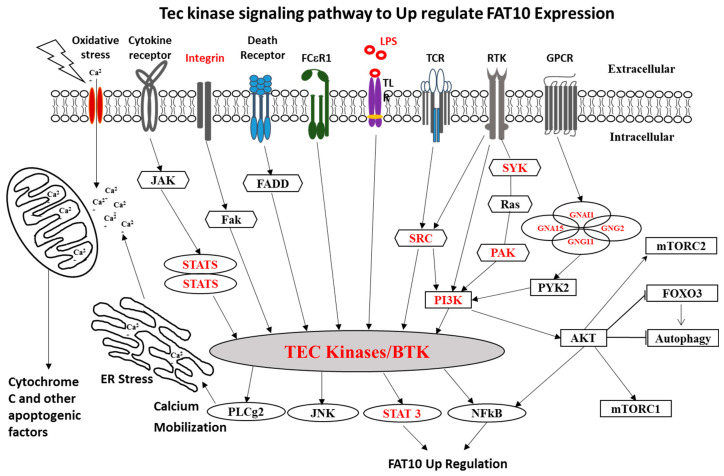
Schematic diagram of Tec kinase signaling and FAT10 pathway. Schematic diagram of Tec kinase signaling activation by oxidative stress and multiple membrane receptors that are involved in a variety of downstream responses, including Ca2+ influx, proliferation, differentiation, and transformation. In alcoholic hepatitis, FAT 10 is the key molecule connected with TEC kinase pathway through STAT3 and NFκB modulations.

There are many gene overexpression pathways of FAT10 which are up-regulated by NFκB activation. Figure 6 summarizes some of the pathways involved. Integrin, LPS, and SYK genes signal through TEC to NFκB and STAT3 [56,57]. The FAT10 gene is overexpressed partly because of a feedback loop involving TNFα and IFNγ that both have NFκB gene present and stimulate the FAT10 expression [58]. TNFα-induced FAT10 expression is dependent on NFκB signaling.

FAT10 is often a potential target gene of STAT3, the expression of which is synergistically induced by NFκB co-stimulation. STAT3 binding stabilizes NFκB on the FAT10 promoter and leads to maximum induction of FAT10 gene expression. Increased FAT10 suppresses the transcriptional activity of the tumor suppressor p53, which accelerates the protein degradation of FAT10. The FAT10-p53 mutual-suppressive regulation is critical in the control of tumorigenesis, as overexpressed FAT10 may be associated with tumorigenesis or tumor progression in the solid tumor model. The transcriptional synergy by STAT3 and NFκB enhances FAT10 activity to modulate the mutually suppressive loop of FAT10-p53 and thus is associated with tumorigenesis under inflammatory conditions [59].

The inflammation-mediated FAT10 was shown to be transcriptionally regulated by and also to regulate the nuclear factor-κB (NFκB) signaling pathway. Low-dose IFNγ with TNFα synergistically elevated FAT10 expression; preincubation of macrophages with IFNγ strongly enhanced TNFα-induced FAT10 expression. A double enhancement mechanism of TNFα signaling was reported by Michal Kandel-Kfir’s team [60]. IFNγ rapidly induced the expression of TNFα and tumor necrosis factor receptor 1 (TNFR1), which further enhanced the production of TNFα and TNFR1 expression by TNFα. Importantly, IFNγ-induced FAT10 in macrophages from TNFα-deficient or TNFR1-deficient mice was completely suppressed, compared to macrophages from wild type (WT) mice. IFNγ potentiates the TNFα/TNFR1 signaling pathway to induce FAT10 expression in mouse macrophages, mediated through the NFκB network [60].

**Figure 7:**
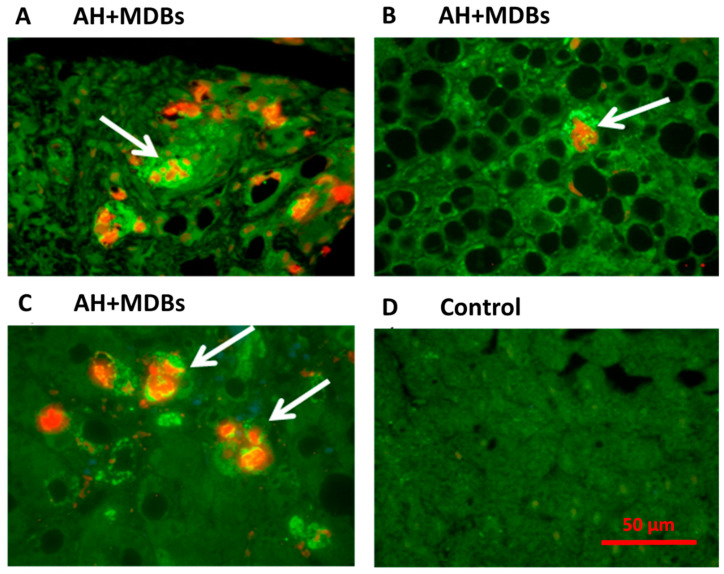

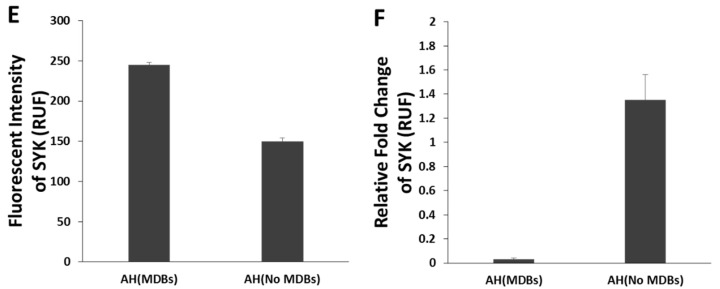
The changes of SYK protein in alcoholic hepatitis and control liver specimen. Liver sections from different patients with AH (**A**–**C**) and control (**D**) were double stained with antibodies to SYK (green) and UB (red). The ballooned hepatocytes (white arrows) stained positive for both SYK in the cytoplasm and UB in the MDB (×741). (**E**) The comparison of morphometric measurements of SYK present in the cytoplasm of MDB forming hepatocytes and in those had not formed MDBs (*p* = 0.001). (**F**) SYK has significantly higher expression in AH patients compared to the control samples by quantitative PCR (*p* = 0.016). [57].

Antibody specific immunofluorescence staining of the liver samples from AH patients shows that SYK is almost exclusively localized in the cytoplasm of the hepatocytes with “balloon cell” appearance which often contain MDBs. This finding suggests that SYK remains where it was expressed by stabilization of the protein (Figure 7A–F). SYK is a protein with 10 phosphorylation sites which are very susceptible to be phosphorylated and activated. SYK also participates in immune cell-signaling pathways and is involved in liver fibrosis development [58]. Potential therapeutic methods targeting the FAT10-related molecular pathways, such as NF-κB/STAT3 signaling, as well as targeting the FAT10 conjugation pathway, seem to be promising as the genetically modified FAT10 knockout mice show less severe phenotypes even with NF-κB/STAT3 signaling activation [61]. As mentioned above, the Tec kinase signaling pathway connects the TLR/NFKB/CXCR4/7 and PI3K/AKT/mTORC1 signaling pathways together in Mallory–Denk body (MDB) formation in AH. The molecular mechanisms of FAT10 in MDB formation and AH pathogenesis is complicated; more detailed studies are needed to confirm the role of FAT10 and MDB formation in hepatocellular carcinoma development.

## 4. Treatment for Alcoholic Hepatitis

As FAT10 was only found in human and mouse liver specimens and plays a critical role in the development of alcoholic hepatitis, FAT10 overexpression has been treated experimentally. Silibinin, a natural flavanone, derived from the milk thistle plant (Silybum marianum), was illustrated for several medicinal uses such as liver-protective, anti-oxidant, anti-cancer, anti-inflammation, and many others [62]. Using flavanone derivatives of milk thistle, when taken orally, inhibit IFNγ, TNFα, NFκB, and STAT3, which inhibits the expression of FAT10 [63]. However, silibinin’s clinical applications and therapeutic efficiency are limited due to its low water solubility, leading to poor absorbance and bioavailability. The combination of silibinin with phosphatidylcholine (PC) as a formulation may enhance the solubility and then markedly enhance bioavailability and therapeutic efficiency [62]. Silibinin has been shown to inhibit many cell-signaling pathways in preclinical models, demonstrating promising effects against liver disorders and cancer through in vitro and in vivo studies. Polachi et al. summarized the pharmacokinetic properties, bioavailability, safety data, clinical activities, and modulatory effects of silibinin in different cell-signaling pathways against liver disorders and cancer [62].

During AH, FAT10 is up-regulated (6.5–7 fold) and NFκB is the key nodal hub of the IFNγ/TNFα-response genes. Silibinin is reported to be a powerful antioxidant and has anti-carcinoma effects against various carcinomas by affecting various molecular signaling pathways, including IFNγ/TNFα-induced tumor growth [63]. Use of Silibinin and other milk thistle derivatives were studied in treating ALD. In general, FAT10 is overexpressed in cells which share common pathways represented by 35 genes, which involve infectious disease, antimicrobial/inflammatory response, and cell death and survival, while TNFα and NFκB represent the major key nodal molecules in these networks [63].

STAT3 is constitutively activated in many different types of cancer and plays a pivotal role in tumor growth and metastasis. Retrospective studies have showed that STAT3 activation or phospho-STAT3 (pSTAT3, activated forms of STAT3) indicate poor prognosis for many cancers. Silibinin has been shown to inhibit multiple cancer cell-signaling pathways in preclinical models, demonstrating promising anticancer effects in vitro and in vivo. Silibinin may inhibit STAT3 phosphorylation/activation in preclinical cancer models. Several ongoing clinical trials are trying to explore the role of silibinin in cancer [64]. The research data support the use of milk thistle derivatives in a clinical trial for the treatment of AH.

## 5. Conclusions

In the liver, FAT10 is essential to maintain the function of cell protein quality control and Mallory–Denk body (MDB) formation. FAT10 overexpression in AH leads to balloon degeneration and MDB aggregation formation through modulating 26s proteasome’s proteases. KO mice fed DDC failed to develop MDBs which suggests that lack of FAT10, like SAMe and Betaine feeding, may suppress MDBs’ formation and attenuate the progression of AH. FAT10 functions not only as a ubiquitin independent and transferable signal for degradation by the proteasome, but also as an interactor with the SUMO system. FAT10 may affect the development of alcoholic liver disease via interfering alcohol-mediated up-regulation of SUMOylation. In alcoholic hepatitis, TNFα/IFNγ induces FAT10 expression via NFκB and/or STAT3 pathways. NFκB is the key nodal hub of the IFNγ/TNFα-response genes. As the inflammation in the hepatocytes is a critical transformation from simple liver steatosis to steatohepatitis, FAT10 and related TNFα/IFNγ changes via NFκB and/or STAT3 pathways could be the candidate molecular parameters for low-dose alcohol effects on alcoholic hepatitis. The interferon sequence response element (ISRE) is located on the FAT10 promoter and responds to IFNγ and TNFα to induce the immunoproteasome catalytic subunits LMP2 and LMP7 colocalizing with FAT10/ubiquitin to modulate the formation of MDB. In summary, FAT10 was only found in human and mouse liver specimens and plays critical role in the development of alcoholic hepatitis via NFκB and/or STAT3 pathways induced by TNFα/IFNγ.

Based on the mice and human studies, potential therapeutic targeting FAT10-activating pathways, such as NFκB/STAT3-signaling, as well as targeting the FAT10 conjugation pathway, seem to be promising. Flavanone derivatives of milk thistle may be able to treat AH due to its inhibiting TNFα/IFNγ, NFκB and STAT3 and then suppression of the expression of FAT10. More experiments and clinical trials are needed to achieve this goal.

## Figures and Tables

**Table 1:** Patients with alcoholic hepatitis studied for changes in the expression of FAT10.

#	Fat Macro	Fat Micro	PMN	Lymp	Other Findings	Fibrosis Stage	Duct Metaplasia	MDBs
01-003	2+	1+	0	4+	0	4+	positive	2+
01-011	1+	0	0	0	0	4+	positive	1+
01-015	3+	1+	1+	1+	0	4+	positive	3+
01-016	4+	0	3+	1+	bile thrombi, EM	4+	positive	4+
01-017	2+	0	2+	4+	PMN satellitosis	4+	positive	4+
01-019	4+	0	0	1+	0	4+	positive	3+
01-021	0	0	1+	4+	EM apoptosis	4+	positive	1+
03-001	1+	0	3+	4+	bile thrombi, EM	4+	positive	4+
03-005	2+	0	0	4+	EM	4+	positive	1+
03-006	1+	0	4+	1+	satellitosis, EM	4+	positive	4+
03-007	1+	0	0	2+	0	4+	positive	1+
03-012	2+	0	3+	3+	best satellitosis, EM	4+	positive	4+
03-014	2+	0	2+	0	most MDBs	4+	positive	4+
03-015	4+	0	1+	0	0	4+	positive	2+
03-017	3+	0	2+	3+	EM	3+	positive	1+
03-018	4+	0	4+	2+	satellitosis	4+	positive	3+
03-019	3+	0	3+	0	satellitosis, EM	4+	positive	4+
03-020	1+	0	3+	0	satellitosis	4+	positive	1+
03-022	3+	1+	0	2+	autophagy, EM	4+	positive	3+
03-023	1+	0	4+ satellitosis	1+	autophagy of MDBs, EM	4+	positive	4+
03-024	4+	1+	3+	1+	PMN lymphocytes, EM	4+	positive	4+
03-025	4+	1+	1+	1+	autophagy, EM	3+	positive	3+
03-027	1+	0	4+	4+	PMN satellitosis	4+	positive	4+

Fat Macro: fat macrovesicular deposition; fat Micro: fat microvesicular deposition; PMN: polymorphonuclear leukocyte; lymp: lymphocytes; MDB: Mallory–Denk body.
